# Bidet toilet use and incidence of hemorrhoids or urogenital infections: A one-year follow-up web survey

**DOI:** 10.1016/j.pmedr.2017.02.008

**Published:** 2017-02-16

**Authors:** Teppei Kiuchi, Keiko Asakura, Makiko Nakano, Kazuyuki Omae

**Affiliations:** a Department of Preventive Medicine and Public Health, School of Medicine, Keio University, 35 Shinanomachi, Shinjuku-ku, Tokyo 160-8582, Japan; b Department of Environmental and Occupational Health, School of Medicine, Toho University, Japan

**Keywords:** OR, odds ratio, CI, confidence interval, SES, socioeconomic status, Bidet toilet, Hemorrhoids, Female urogenital infection

## Abstract

Although bidet toilets are widely used in Japan, the relationship between habitual bidet toilet use and the incidence of hemorrhoids or urogenital infections has not been prospectively studied.

We performed a web survey and followed bidet toilets users and non-users to assess the incidence of hemorrhoids or urogenital infections from 2013 to 2014. Study subjects were randomly selected from a research company's (Macromill, Inc.) web panel. The baseline survey inquired about toilet use and confounding parameters, and the follow-up survey examined outcome parameters.

A total of 7637 subjects were analyzed using single or multiple logistic regression models. The prevalence odds ratios (ORs) between bidet toilet users and non-users for hemorrhoids, urological infections, and vulval pruritus were significantly > 1.0 but their incidence ORs were not significant. The adjusted incidence OR for bacterial vaginitis symptoms was significant (2.662, 95% confidence interval [CI] [1.315–5.520]).

These findings suggest that positive relations between habitual bidet toilet use and hemorrhoids and urogenital symptoms, except bacterial vaginitis, were due to reverse causation. The incidence of bacterial vaginitis might be caused by bidet toilet use, but the incidence rates were too small to make a definite conclusion, and further studies are needed.

## Highlights

• Habitual bidet toilet use might cause bacterial vaginitis, but not conclusive.
• Hemorrhoids and other urogenital infections are not caused by bidet toilet use.
• The positive correlations reported earlier seem due to reverse causation.

## 1 Introduction

A bidet toilet is a sanitary toilet facility with a warm lavatory seat and a warm water shower to clean the anal or urogenital area after defecation, urination, or menstruation. In Japan, the penetration rate of the bidet toilet use in general households was approximately 77.5% (Cabinet Office, 2015) and it will become popular not only in Eastern but Western countries in the future to improve a quality of life in a rest room.

Some physicians have expressed concern that the habitual use of a bidet toilet causes hemorrhoids or urogenital infection (Ogino et al., 2010, Ogino, 2010, Kohdaira, 2009). However, these studies failed to exclude reverse causation, and the role of habitual bidet toilet use as a cause of hemorrhoids or urogenital infections remains controversial.

Here, to assess the relationship between habitual bidet toilet use and the incidence of hemorrhoids or urogenital infection, we conducted a web survey. A baseline survey was performed in February 2013, and a follow-up survey in February 2014.

## 2 Materials and methods

This study was approved by the Ethical Committee, Keio University School of Medicine (approval number 20120410).

### 2.1 Study subjects

The study subjects were selected from among approximately one million people anonymously registered with a leading Japanese website research company's (Macromill, Inc.) web panel. A total of 18,562 people were randomly selected using a computer program, to whom a web survey questionnaire was randomly delivered until the number of respondees exceeded 10,000. A total of 10,305 individuals were involved in the baseline survey.

A follow-up web survey was conducted in February 2014. Among the 10,305 baseline survey subjects, 8255 subjects participated again, whereas 2050 subjects did not reply to the follow-up survey (follow-up rate 80.1%). Of these followed subjects, 618 met exclusion criteria, which included inconsistent answers about “bidet toilet use” at the baseline and follow-up surveys (*n* = 407), age older than 80 years (*n* = 103), abnormal frequency of daily urination (< 3 or > 15) (*n* = 46), abnormal hours of daily sleep (< 4 h) (*n* = 22), females older than 60 years with current menstruation (*n* = 22), and unusually prolonged bidet toilet use (> 180 s) (*n* = 15). The final number of subjects analyzed was 7637. To discuss subject characteristics, we compared the proportion of analyzed subjects against Japanese National Survey data (Ministry of Internal Affairs and Communications, 2013).

### 2.2 Exposure, outcome, and confounding parameters

Bidet toilet use as an exposure parameter was queried in both the baseline and follow-up surveys. The frequency of bidet toilet use was scored as “never used”, “use less than once a week”, “use every day or more than once a week”. For statistical analysis, we defined subjects answering at baseline “never use” or “use less than once a week” as “non-habitual users” and those answering “use every day or more than once a week” as “habitual users.”

Outcome parameters surveyed were physician diagnosis and subjective symptoms of hemorrhoids, irritated perianal skin, cystitis, pyelonephritis, candida vaginitis, bacterial vaginitis, and vulval pruritus. Subjective symptoms of each disease are highly specific, and usually used as important clues to diagnose these diseases. To avoid misunderstanding or confusion of the outcome questions, signs and symptoms of the outcomes were displayed on the same screen as the questions. For example, for candida vaginitis, “If you contract candida vaginitis, your vaginal discharge contains white/yellow-green gloppy or clumpy substance resembling sake paste, cottage cheese, yogurt, or tofu draff, and you suffer from an intensely-itching in the area of the vulva or vagina.” We asked about past history at the baseline survey and about the outcome parameters at the follow-up survey. In the follow-up survey, we requested the subject to answer whether “(1) Newly diagnosed (or experienced symptoms) during the period from the baseline survey to the follow-up survey (February 2013 to February 2014),” “(2) Ever diagnosed (or experienced symptoms) before the baseline survey (before January 2013),” or “(3) Never diagnosed (or experienced symptoms).” If the subject selected the first answer, we counted him/her as an incidence case and if he/she selected the second answer, we counted him/her as a prevalence case.

Other questions asked at baseline included questions about smoking, drinking, fitness, sleeping, showering/bathing, bowel movements, direction of wiping the anus after defecation, menstrual status, sexual activity, academic background, and past/current histories of diseases. Basic characteristics of the subjects such as age, sex, residential area, etc. were already registered in the website research company records, and were provided to us.

### 2.3 Statistical analysis

Conditions between habitual and non-habitual users were compared using the chi-squared test.

The prevalence and incidence of outcomes were assessed using a crude or adjusted ORs applying a single or a multiple logistic regression model. Before adopting the multiple logistic regression models, a univariate analysis between hemorrhoids and cystitis, pyelonephritis and vaginitis and each possible confounding parameter was conducted. A list of *p* values applying 2-by-2 or 2-by-3 table analysis between the outcome and the parameters is shown in Supplemental Table 1. Consequently, as explanatory variables in the multiple logistic regression model, we selected such confounding factors as age category (20–39/40–59/60–79 years), marital status, educational background (high/low), smoking habits (current/former/non), alcohol drinking habits (habitual/occasional/non), current history of immune-related diseases (yes/no), and current constipation (yes/no) for analysis of hemorrhoids and related outcomes; and age category, marital status, smoking habit, current menstrual status (yes/no), sexual activity (yes/no) and current constipation (yes/no) for analysis of urogenital outcomes.

Statistical significance was assessed by a two-tailed analysis with p < 0.05 considered significant. All statistical analyses were performed using commercial software (JMP version 10.0.2®; SAS Institute, Cary NC, USA).

## 3 Results

Among 7637 subjects, 4272 (55.9%) were classified as “habitual users” and 3365 (44.1%) as “non-habitual users.” The proportion of habitual users was larger in males than females. Compared to non-habitual users, habitual users were more likely to be old, married, wealthy, and have a lower constipation rate (female), a higher menopausal rate (female) and higher sexual activity (female). Some confounding parameters showed statistically significant rates, but the differences in the rates between the habitual and non-habitual users were small (Table 1).

Table 2 shows the prevalence and incidence ORs of hemorrhoids diagnosed by a physician, subjective symptoms of hemorrhoids, and subjective symptoms of irritated perianal skin by sex. Both crude and adjusted prevalence ORs of these three disease/symptoms were significantly > 1 in both male and female habitual users. In contrast, the crude and adjusted incidence ORs did not show any significance.

Table 3 shows prevalence and incidence ORs of urogenital outcomes diagnosed by a physician and subjective symptoms of the outcomes in female subjects.

Both a physician diagnosis and subjective symptoms of urological infections, namely, cystitis and pyelonephritis, showed significantly higher crude prevalence ORs in habitual users, but nonsignificant ORs for crude incidence.

Neither prevalence nor incidence ORs of candida vaginitis were statistically significant in habitual users with regard to either physician diagnosis or subjective symptoms.

Both crude and adjusted prevalence ORs of vulval pruritus were significantly higher in habitual users, but significance was lost in the incidence ORs.

The adjusted prevalence OR of bacterial vaginitis symptoms was just failed to reach statistical significance (95% CI = [0.998–2.084]), and the adjusted incidence OR was significant.

The adjusted incidence ORs of other confounding parameters are shown in Supplemental Table 2a, b and c.

## 4 Discussion

In this prospective 1-year follow-up study of bidet toilet users, we found that hemorrhoids and urogenital infections, excluding bacterial vaginitis, were not causally related to habitual bidet toilet use. Although the incidence of bacterial vaginitis might have been caused by bidet toilet use, incidence rates were small and further studies are needed.

The most significant findings in this study are in its prospective 1-year follow-up of bidet toilet users and incidence calculation. Except for bacterial vaginitis, most point estimates of the crude or adjusted prevalence ORs of all hemorrhoid-related and urogenital outcomes were larger than those of the crude and adjusted incidence ORs. Further, statistical significance in the crude and adjusted prevalence ORs disappeared in the crude and adjusted incidence ORs. These findings strongly suggest that the positive correlations between the urogenital outcomes and habitual bidet toilet use reported earlier (Ogino et al., 2010, Ogino, 2010, Kohdaira, 2009) were not causal relationships, but rather might have been reverse causation. In general, persons with discomfort around the anal or genital areas may prefer to use a bidet toilet.

The incidence ORs of bacterial vaginitis were inversely associated with the prevalence ORs compared to other outcomes, and the adjusted incidence OR of subjective symptoms of bacterial vaginitis was statistically significant. Since the incidence rates of a physician diagnosis and subjective symptoms of bacterial vaginitis in the habitual users were not enough (0.4% and 1.2%) to conclude a causal relationship, these results require additional investigation and long-term follow-up.

Outcome parameters in this study were collected using web questionnaires. The information on subjective symptoms could reveal pre-diagnosed disease but was susceptible to subjects' understanding, sensitivity, and recall. To avoid connecting answers for bidet toilet use with answer for disease diagnosis/symptoms, the questions about toilet use were located last in the follow-up survey and were compared with the answers in the baseline survey.

This study recruited subjects from a web-based registered commercial panel. It is known that samples collected with Internet-based approaches generally tend to be younger and have a higher SES than traditional, non-Internet-based samples (Hays et al., 2015, Remillard et al., 2014, Yasunaga et al., 2006). Comparison between analyzed subjects and the national population also showed that the analyzed subjects did not represent the overall Japanese population, and characteristics slightly differed between the analyzed and unanalyzed subjects. Age distribution was skewed to an older age group. Even though subject propensity to respond was potentially related to strong concern about the study topic, namely bidet toilet use and/or related diseases/symptoms, this kind of selection is not specific to web surveys. Because household income and residential area were not related to outcomes, and OR analyses were adjusted for age distribution, marital status, and sexual activity, the internal validity of this study is unlikely to have been impaired.

Web-based surveys can help make data collection more precise in one way. The electronic data handling process averts errors in the data entry and coding process. Real-time questionnaire administration with validation checks inhibits incomplete answers. Gelder et al. also argued that the anonymity of web-based surveys allows traditional epidemiologic risk factors to be collected with equal or even better reliability due to the anonymity and private respondent feelings of web-based surveys (van Gelder et al., 2010). This study, which dealt with a rather personal topic, might have strongly benefited from being conducted as a web survey.

Information bias about exposure and possible confounding parameters might have been small because these parameters were collected at the baseline study. In contrast, a degree of recall bias with regard to outcome parameters collected at the follow-up study might have been unavoidable because we asked the questions one year after the baseline study. In general, habitual users may be inclined to better remember the relationship between the use of a bidet toilet and outcomes. Some authors have noted that higher-SES respondents contacted by web surveys may be more aware of, and better able to identify symptoms (Remillard et al., 2014). However, except for bacterial vaginitis, the differences in incidence rates between the habitual and non-habitual users were small and the incidence ORs were not significant. Accordingly, this bias is unlikely to have had any major impact.

Characteristics of the 7637 analyzed subjects and 2668 unanalyzed (lost to follow-up survey [2050], or removed [618]) subjects are shown in Supplemental Table 3. It should be noted that subjects lost to follow-up were more likely to be non-habitual, younger, unmarried subjects and subjects with a higher rate of sexual activity. Supplemental Table 3 also shows the Japanese National Survey data; comparison of our present subjects to the National Survey population shows that the proportions of sex and marital status were similar, but that age distribution was biased toward older people, and residence was skewed to the Kanto region (near the Tokyo metropolitan area) and Kinki region (near the Osaka metropolitan district). Also, the proportion of middle-income households (4–8 million yen) was larger. Since residential area and household income were not related to outcomes, we consider it unlikely that they influence the generalizability of this study. Subgroup analysis showed that the relation between habitual bidet toilet use and the incidence of hemorrhoids and urological infection was weaker in younger subjects. The relation with vaginal infection tended to be greater, but incidence rates were not large. Accordingly, the external validity of this study is unlikely to have been impaired.

Several potential biases that were not adjusted in this study should be noted. The presence of hemorrhoids or urogenital infections may have affected bidet toilet use and may have enhanced disease recall/symptom recognition. Patients asked about symptoms in the year after the first survey may have had enhanced recall compared with patients asked to recall prior symptoms in the first survey.

Since this study asked general population with simple questionnaire, we didn't ask about the place subjects used bidet toilets or detailed functions of toilets. To reveal whether these parameters affected relationships or not needs further studies.

In summary, we found that hemorrhoids and urogenital infections, except bacterial vaginitis, were not causally related to habitual bidet toilet use. The incidence of bacterial vaginitis might be caused by the bidet toilet use, but incidence rates were low and further studies are needed.

## Funding

This study was sponsored by the Japan Sanitary Equipment Industry Association (JSEIA). JSEIA had no role in the design of the study, the collection and analysis of the data, or the preparation of the manuscript.

## Conflict of interest

This study was sponsored by JSEIA.

## Ethical approval

All procedures performed in studies involving human participants were in accordance with the ethical standards of the institutional and/or national research committee and with the 1964 Helsinki declaration and its later amendments or comparable ethical standards. This article does not contain any studies with animals performed by any of the authors. This study was approved by the Ethical Committee, Keio University School of Medicine (approval number 20120410).

## Informed consent

Informed consent was obtained from all individual participants included in the study.

## Transparency document

Transparency document.

## Figures and Tables

**Table 1:** Characteristics of 7637 subjects.

	All subjects			Female			Male		
Habitual use of Bidet toilet	Yes	No	p^a^	Yes	No	p^a^	Yes	No	p^a^
	n(%)	n(%)		n(%)	n(%)		n(%)	n(%)	
N of subjects	4272(55.9)	3365(44.1)		1983(51.3)	1882(48.7)		2289(60.7)	1483(39.3)	

Age distribution (years)									
20–29	177(24.7)	539(75.3)	⁎⁎⁎	51(15.2)	284(84.8)	⁎⁎⁎	126(33.1)	255(66.9)	⁎⁎⁎
30–39	427(36)	759(64)		166(29.1)	405(70.9)		261(42.4)	354(57.6)	
40–49	590(46.8)	671(53.2)		217(35.7)	391(64.3)		373(57.1)	280(42.9)	
50–59	816(63.0)	480(37)		371(58.8)	260(41.2)		445(66.9)	220(33.1)	
60–69	1145(67.7)	547(32.3)		635(65.1)	340(34.9)		510(71.1)	207(28.9)	
70–79	1117(75.2)	369(24.8)		543(72.9)	202(27.1)		574(77.5)	167(22.5)	

Marriage status									
Married	3289(62.2)	1999(37.8)	⁎⁎⁎	1521(55.5)	1221(44.5)	⁎⁎⁎	1768(69.4)	778(30.6)	⁎⁎⁎
Unmarried	983(41.8)	1366(58.2)		462(41.1)	661(58.9)		521(42.5)	705(57.5)	

Educational background^b^, ^d^									
Low	1800(54.7)	1489(45.3)		1007(52.7)	904(47.3)		793(57.5)	585(42.5)	⁎⁎
High	2442(56.8)	1856(43.2)		958(49.8)	964(50.2)		1484(62.5)	892(37.5)	

Household income (million yen/yr)^d^									
< 4	1260(50.9)	1214(49.1)	⁎⁎⁎	639(48.5)	678(51.5)	⁎⁎⁎	621(53.7)	536(46.3)	⁎⁎⁎
4– < 8	1671(58.7)	1175(41.3)		733(53.4)	640(46.6)		938(63.7)	535(36.3)	
≥ 8	865(67.9)	408(32.1)		343(62.9)	202(37.1)		522(71.7)	206(28.3)	

Cigarette smoking									
Never	2788(55.0)	2285(45.0)	⁎⁎⁎	1656(52.5)	1496(47.5)	⁎⁎⁎	1132(58.9)	789(41.1)	⁎⁎⁎
Former	781(63.8)	443(36.2)		156(51.0)	150(49.0)		625(68.1)	293(31.9)	
Current	703(52.5)	637(47.5)		171(42.0)	236(58.0)		532(57.0)	401(43.0)	

Alcohol drinking									
No	1033(52.8)	923(47.2)	⁎⁎⁎	663(51.4)	627(48.6)	⁎	370(55.6)	296(44.4)	⁎⁎⁎
< once/week	1496(51.6)	1404(48.4)		792(49.3)	814(50.7)		704(54.4)	590(45.6)	
≥ once/week	1743(62.7)	1038(37.3)		528(54.5)	441(45.5)		1215(67.1)	597(32.9)	

Immune-suppressing diseases^c^									
Yes	433(67.0)	213(33.0)	⁎⁎⁎	153(61.4)	96(38.6)	⁎⁎⁎	280(70.5)	117(29.5)	⁎⁎⁎
No	3839(54.9)	3152(45.1)		1830(50.6)	1786(49.4)		2009(59.5)	1366(40.5)	
Frequency of showering/bathing^d^									
≥ once/day	3262(55.5)	2618(44.5)	⁎	1509(50.0)	1509(50.0)	⁎⁎	1753(61.3)	1109(38.7)	
< once/day	867(58.8)	608(41.2)		393(56.2)	306(43.8)		474(61.1)	302(38.9)	

Current constipation									
Yes	1769(51.6)	1660(48.4)	⁎⁎⁎	969(46.4)	1118(53.6)	⁎⁎⁎	800(59.6)	542(40.4)	
No	2503(59.5)	1705(40.5)		1014(57.0)	764(43.0)		1489(61.3)	941(38.7)	

Current menstruation^d^									
Yes				516(31.7)	1114(68.3)	⁎⁎⁎			
No				1458(65.6)	764(34.4)				

Direction of wiping anus after defecation^d^									
Front to back				1121(49.2)	1156(50.8)	⁎⁎			
Back to front				553(43.8)	711(56.3)				

Sexual activity (≥ once/year)^d^									
Yes				1358(56.4)	1049(43.6)	⁎⁎⁎			
No				524(42.6)	705(57.4)				

⁎ p < 0.05.

⁎⁎ p < 0.01.

⁎⁎⁎ p < 0.001.

a Chi-square test.

b Low means those graduated from high school or lower schools. High means those graduated from junior college or higher educational institutions.

c Diabetes, malignancy and immune disorder.

d Some subjects did not answer the questions or selected “Unknown”. Numbers of such subjects were 50 in “Educational background”, 1044 in “Household income”, 282 in “Frequency of showering/bathing”, 13 in “Current menstruation”, 324 in “Direction of wiping anus after defecation”, and 229 in “Sexual activity”.

**Table 2:** Prevalence rates, incidence rates, and odds ratios (ORs) of habitual bidet toilet use in hemorrhoid and related symptom.

		Yes (%)	No (%)	Prevalence odds ratio		Yes (%)	No (%)	Incidence odds ratio	
				Crude (95%CI)	Adjusted (95%CI)			Crude (95%CI)	Adjusted (95%CI)
Doctor's diagnosis of hemorrhoid									
Male	Habitual user	648(28.3)	1641	2.407(2.026–2.859)	1.788(1.492–2.148)	17(1.0)	1624	0.823(0.414–1.635)	1.013(0.489–2.106)
	Non-habitual user	209(14.1)	1274	1	1	16(1.3)	1258	1	1
Female	Habitual user	395(19.9)	1588	1.479(1.248–1.752)	1.465(1.220–1.762)	22(1.4)	1566	1.400(0.733–2.677)	1.923(0.950–3.971)
	Non-habitual user	271(14.4)	1611	1	1	16(1.0)	1595	1	1

Subjective symptom of hemorrhoid									
Male	Habitual user	1302(56.9)	987	1.714(1.502–1.955)	1.519(1.319–1.749)	89(9.0)	898	0.890(0.650–1.217)	1.117(0.799–1.562)
	Non-habitual user	645(43.5)	838	1	1	84(10.0)	754	1	1
Female	Habitual user	953(48.1)	1030	1.165(1.027–1.322)	1.318(1.147–1.515)	69(6.7)	961	0.632(0.461–0.867)	1.058(0.740–1.508)
	Non-habitual user	833(44.3)	1049	1	1	107(10.2)	942	1	1

Subjective symptom of irritated skin around anus									
Male	Habitual user	1212(52.9)	1077	1.680(1.471–1.917)	1.618(1.406–1.864)	152(14.1)	925	1.094(0.843–1.418)	1.267(0.958–1.681)
	Non-habitual user	595(40.1)	888	1	1	116(13.1)	772	1	1
Female	Habitual user	867(43.7)	1116	1.144(1.007–1.300)	1.222(1.062–1.406)	134(12.0)	982	0.780(0.611–0.995)	1.024(0.80–1.345)
	Non-habitual user	761(40.4)	1121	1	1	167(14.9)	954	1	1

95%CI: 95% confidence interval. Adjusted OR: odds ratio adjusted by confounders (see text) using logistic regression model.

**Table 3:** Prevalence rates, incidence rates, and odds ratios (ORs) of habitual bidet toilet use in urogenital outcomes.

		Yes(%)	No(%)	Prevalence odds ratio		Yes (%)	No (%)	Incidence odds ratio	
				Crude (95%CI)	Adjusted (95%CI)			Crude (95%CI)	Adjusted (95%CI)
Doctor's diagnosis of:									
Cystitis									
Habitual user		779(39.3)	1204	1.376(1.205–1.570)	1.092(0.942–1.266)	34(2.8)	1170	1.065(0.658–1.725)	0.818(0.480–1.395)
Non-habitual user		602(32.0)	1280	1	1	34(2.7)	1246	1	1
Pyelonephritis									
Habitual user		189(9.5)	1794	1.494(1.180–1.891)	1.115(0.862–1.446)	4(0.2)	1790	0.559(0.163–1.913)	0.507(0.127–1.794)
Non-habitual user		124(6.6)	1758	1	1	7(0.4)	1751	1	1
Candida vaginitis									
Habitual user		393(19.8)	1590	1.045(0.891–1.225)	1.125(0.942–1.345)	9(0.6)	1581	0.341(0.159–0.733)	0.814(0.330–1.817)
Non-habitual user		360(19.1)	1522	1	1	25(1.6)	1497	1	1
Bacterial vaginitis									
Habitual user		47(2.4)	1936	1.038(0.683–1.578)	1.344(0.844–2.152)	8(0.4)	1928	1.268(0.439–3.660)	2.679(0.866–8.678)
Non-habitual user		43(2.3)	1839	1	1	6(0.3)	1833	1	1
Vulval pruritus									
Habitual user		50(2.5)	1933	1.255(0.819–1.923)	1.074(0.676–1.720)	5(0.3)	1928	0.681(0.216–2.148)	1.001(0.276–3.415)
Non-habitual user		38(95.4)	1844	1	1	7(0.4)	1837	1	1

Subjective symptoms of:									
Cystitis									
Habitual user		878(44.3)	1104	1.322(1.162–1.503)	1.105(0.956–1.276)	53(4.8)	1052	0.833(0.575–1.206)	0.871(0.573–1.316)
Non-habitual user		707(99.8)	1175	1	1	67(5.7)	1108	1	1
Pyelonephritis									
Habitual user		164(8.3)	1819	1.511(1.173–1.945)	1.077(0.820–1.420)	6(0.3)	1813	0.976(0.314–3.033)	0.870(0.235–3.117)
Non-habitual user		106(5.6)	1776	1	1	6(0.3)	1770	1	1
Candida vaginitis									
Habitual user		450(22.7)	1533	0.971(0.835–1.128)	1.126(0.951–1.333)	23(1.5)	1510	0.596(0.352–1.011)	1.325(0.706–2.424)
Non-habitual user		437(23.2)	1445	1	1	36(2.5)	1409	1	1
Bacterial vaginitis									
Habitual user		81(4.1)	1902	1.230(0.879–1.720)	1.438(0.998–2.084)	22(1.2)	1880	1.407(0.728–2.721)	2.662(1.315–5.520)
Non-habitual user		63(3.3)	1819	1	1	15(0.8)	1804	1	1
Vulval pruritus									
Habitual user		221(11.1)	1762	1.323(1.069–1.637)	1.303(1.032–1.649)	33(1.9)	1729	0.577(0.373–0.894)	0.803(0.491–1.298)
Non-habitual user		163(8.7)	1719	1	1	55(3.2)	1664	1	1

95%CI: 95% confidence interval. Adjusted OR: odds ratio adjusted by confounders (see text) using logistic regression model.

## Appendix A Supplementary data

Supplementary tables Tables showing Univariate analysis between outcome parameters and possible confounding factors, Adjusted OR of risk parameters in incidence analyses, and Characteristics of subjects analyzed and unanalyzed. Image 1

## References

[bb0005] Cabinet Office (2015). Consumer Confidence Survey. http://www.esri.cao.go.jp/jp/stat/shouhi/shouhi.html.

[bb0010] van Gelder M.M., Bretveld R.W., Roeleveld N. (2010). Web-based questionnaires: the future in epidemiology?. Am. J. Epidemiol..

[bb0015] Hays R.D., Liu H., Kapteyn A. (2015). Use of Internet panels to conduct surveys. Behav. Res. Methods.

[bb0020] Kohdaira T. (2009). Correlation of recurrent cystitis and using of toilet seat with shower unit. Jpn. J. Urol. Surg..

[bb0025] Ministry of Internal Affairs and Communications (2013). Statistics of Japan. http://www.stat.go.jp/data/nihon/back13/02.htm.

[bb0030] Ogino M. (2010). Relationship between habitual use of warm-water cleaning toilets and aggravation of vaginal microflora. Perinat. Care.

[bb0035] Ogino M., Iino K., Minoura S. (2010). Habitual use of warm-water cleaning toilets is related to the aggravation of vaginal microflora. J. Obstet. Gynaecol. Res..

[bb0040] Remillard M.L., Mazor K.M., Cutrona S.L., Gurwitz J.H., Tjia J. (2014). Systematic review of the use of online questionnaires of older adults. J. Am. Geriatr. Soc..

[bb0045] Yasunaga H., Ide H., Imamura T., Ohe K. (2006). Medical research using internet questionnaire in Japan. Nihon Koshu Eisei Zasshi.

