# Supplementary material for: Bidet toilet use and incidence of hemorrhoids or urogenital infections: A one-year follow-up web survey
Source: Prev Med Rep. 2017 Feb 16;6:121–5. doi: 10.1016/j.pmedr.2017.02.008 (PMC5345955; doi:10.1016/j.pmedr.2017.02.008)
Supplement: Supplementary file 1 — Transparency document. [file mmc1.pdf]

ELSEVIER

Preventive Medicine

# Corresponding Author's Declaration Form
Preventive Medicine

Manuscript title:	Bidet toilet use and incidence of hemorrhoids or urogenital infections: a one-year follow-up web survey
Corresponding author:	Teppei Kiuchi
Additional authors in the order provided in the manuscript:	Keiko Asakura, Makiko Nakano, Kazuyuki Omae

The corresponding author must provide statements of authorship, originality, conflicts of interest, and research funding on behalf of all authors of the manuscript.

Authorship and Originality:
The corresponding author warrants that all aforementioned authors fulfill the criteria of authorship as defined by the International Committee of Medical Journal Editors (ICMJE) and explained here. The corresponding author further warrants that the work described in this manuscript has not been published before and is not (nor will be) under consideration elsewhere while under review in *Preventive Medicine*; that all authors approved the present submitted version and their institutions have no objections to the manuscript's contents.

Conflict of interest:
The corresponding author submits the following disclosure of financial or other relationships with companies or organizations that are stakeholders in the topic of the manuscript. For details read this fact sheet. Choose one below:

☑	The authors have no conflicts of interest to disclose
☐	The following authors report specific relationships (name author, nature of the relationship, and company or organization):

Funding source:
All sources of funding that could have influenced or could be perceived to influence the outcome of this work should be acknowledged. You as corresponding author should declare any involvement of study sponsors in the study design; collection, analysis and interpretation of data; the writing of the manuscript; the decision to submit the manuscript for publication. If the study sponsors had no such involvement, this should be stated. Choose one below:

☐	There were no sources of funding that could have influenced the outcome of this work
☑	The following authors report financial support (name author, granting organization, grant number, or company sponsoring the study):<br>This study was sponsored by the Japan Sanitary Equipment Industry Association (JSEIA). JSEIA had no role in the design of the study, the collection and analysis of the data, or the preparation of the manuscript.

The corresponding author signed this statement on behalf of all coauthors to indicate that the above information is true, correct and complete.

Signature: Teppei Kiuchi	Print name: Teppei Kiuchi
	Date: Aug 11 2016

(This form is fillable with adobe software or equivalent. Both graphic and electronic validation signatures via software are acceptable. Alternatively you can sign by hand. Please upload the signed copy with your submission.)
