# Supplementary material for: Bidet toilet use and incidence of hemorrhoids or urogenital infections: A one-year follow-up web survey
Source: Prev Med Rep. 2017 Feb 16;6:121–5. doi: 10.1016/j.pmedr.2017.02.008 (PMC5345955; doi:10.1016/j.pmedr.2017.02.008)
Supplement: Supplementary tables — Tables showing Univariate analysis between outcome parameters and possible confounding factors, Adjusted OR of risk parameters in incidence analyses, and Characteristics of subjects analyzed and unanalyzed. [file mmc2.pdf]

Supplemental Table 1 List of *p* values applying univariate analysis between outcome parameters and possible confounding factors.

	Doctor's diagnosis of:					Subjective symptom of:				
	Hemorrhoid			Candida	Bacterial	Hemorrhoid			Candida	Bacterial
	All	Male	Female	vaginitis	vaginitis	All	Male	Female	vaginitis	vaginitis
< Prevalence analysis >										
Age distribution	<.0001	<.0001	0.1326	<.0001	0.0052	<.0001	<.0001	<.0001	<.0001	0.0033
Marriage status	<.0001	<.0001	0.0028	<.0001	0.4004	<.0001	<.0001	<.0001	<.0001	0.0721
Educational background	0.0534	0.212	0.735	0.309	0.3068	<.0001	0.0028	0.0091	0.0106	0.8337
Household income	0.1464	0.1092	0.7008	0.1589	0.658	0.3819	0.8629	0.6854	0.1418	0.4876
Region of residence	0.8756	0.9166	0.5368	0.034	0.0983	0.2928	0.3213	0.6204	0.1975	0.9254
Cigarette smoking	<.0001	<.0001	0.4081	0.0008	0.077	<.0001	<.0001	0.0147	0.0005	0.027
Alcohol drinking	0.0007	0.0078	0.9765	0.5952	0.4987	<.0001	<.0001	0.0631	0.0354	0.1837
Immune-suppressing diseases	<.0001	<.0001	0.0127	0.2553	0.8241	0.0875	0.0402	0.8707	0.2568	0.8134
Frequency of showering/bathing	0.0799	0.0142	0.1011	0.2615	0.1851	0.1211	0.0067	0.8752	0.2239	0.404
Current constipation	<.0001	0.0002	0.0004	0.0711	0.0285	<.0001	<.0001	<.0001	0.01	0.0013
Current menstruation				<.0001	0.0277				<.0001	0.0027
Direction of wiping anus after defecation				0.0056	0.6881				0.0644	0.1979
Sexual activity				<.0001	0.0009				<.0001	0.0005
< Incidence analysis >										
Age distribution	0.0432	0.1617	0.2083	<.0001	0.0235	<.0001	<.0001	<.0001	<.0001	0.0049
Marriage status	0.0962	0.0099	0.866	0.358	0.2519	<.0001	0.0005	<.0001	0.0005	0.0288
Educational background	0.3099	0.3989	0.5126	0.8636	1	0.0009	0.1102	0.0035	1	0.7416
Household income	0.1196	0.0248	0.9871	0.2151	0.8453	0.6924	0.7606	0.8831	0.3537	0.2125
Region of residence	0.7364	0.8819	0.2466	0.0557	0.0909	0.4847	0.4573	0.8647	0.25	0.6326
Cigarette smoking	0.2107	0.074	0.9962	0.0275	0.0454	0.9616	0.4285	0.2445	0.2326	0.0793
Alcohol drinking	0.877	0.6768	0.8966	0.8353	0.9469	0.008	0.0823	0.1002	0.6852	0.086
Immune-suppressing diseases	0.033	0.4899	0.0188	0.1153	1	0.285	0.3067	0.6001	0.1074	1
Frequency of showering/bathing	0.3713	0.3394	0.0343	0.1819	0.0868	0.4116	0.2153	0.9485	0.6137	0.2897
Current constipation	0.0087	0.0772	0.0514	0.024	1	0.0001	0.0176	0.0005	0.0248	0.0197
Current menstruation				<.0001	0.0311				<.0001	0.0022
Direction of wiping anus after defecation				1	1				1	0.5907
Sexual activity				0.0002	0.021				<.0001	0.0043

Supplemental Table 2 Adjusted OR of habitual bidet toilet use and other risk parameters on outcome parameters in incidence analyses applying multiple logistic regression model

a. Hemorrhoid

		Doctor's diagnosis of:		Subjective symptoms of:			
		Hemorrhoid		Hemorrhoid		Irritated skin around anus	
		Male	Female	Male	Female	Male	Female
Age distribution	Elder/Young	0.593(0.172-1.884)	0.317(0.125-0.797)	0.472(0.289-0.762)	0.333(0.213-0.518)	0.706(0.480-1.039)	0.476(0.336-0.672)
	Middle/Young	1.298(0.549-3.068)	0.585(0.250-1.364)	0.634(0.420-0.952)	0.548(0.362-0.823)	0.782(0.550-1.112)	0.738(0.531-1.026)
Marriage status	Married/unmarried	0.427(0.185-0.965)	1.175(0.575-2.562)	0.722(0.496-1.049)	0.627(0.447-0.883)	0.865(0.630-1.189)	0.908(0.689-1.201)
Educational background	High/Low	1.276(0.616-2.838)	1.147(0.592-2.252)	1.279(0.917-1.803)	1.357(0.974-1.898)	1.075(0.820-1.414)	1.028(0.794-1.331)
Cigarette smoking	Former/Non	1.067(0.425-2.461)	0.952(0.224-2.770)	1.002(0.629-1.559)	1.577(0.879-2.682)	1.125(0.785-1.595)	1.341(0.849-2.056)
	Current/Non	0.259(0.060-0.773)	1.058(0.308-2.769)	0.884(0.593-1.301)	1.004(0.572-1.670)	1.426(1.040-1.947)	0.691(0.425-1.079)
Alcohol drinking	Occasional/Non	1.411(0.529-4.435)	0.734(0.346-1.566)	1.518(0.940-2.528)	1.111(0.760-1.634)	1.606(1.092-2.402)	1.299(0.966-1.753)
	Habitual/Non	1.475(0.546-4.682)	0.788(0.321-1.845)	1.712(1.064-2.845)	1.010(0.644-1.577)	1.334(0.909-1.991)	1.222(0.864-1.725)
Immune-suppressing diseases	Yes/No	1.898(0.675-4.606)	2.940(1.268-6.251)	1.020(0.598-1.660)	1.211(0.703-1.985)	1.227(0.841-1.760)	1.022(0.660-1.534)
Current constipation	Yes/No	1.862(0.922-3.744)	1.953(0.990-4.074)	1.495(1.073-2.071)	1.471(1.060-2.054)	1.767(1.353-2.303)	1.252(0.972-1.615)
Habitual bidet toilet use	Yes/No	1.013(0.489-2.106)	1.923(0.950-3.971)	1.117(0.799-1.562)	1.058(0.740-1.508)	1.267(0.958-1.681)	1.024(0.780-1.345)

b. Urological infection

		Doctor's diagnosis of:		Subjective symptoms of:	
		Cystitis	Pyelonephritis	Cystitis	Pyelonephritis
Age distribution	Elder/Young	1.088(0.384-3.271)	0.113(0.012-1.320)	0.459(0.208-1.041)	0.035(0.001-0.530)
	Middle/Young	1.361(0.612-3.064)	0.791(0.121-7.105)	0.775(0.444-1.335)	1.013(0.164-8.535)
Marriage status	Married/unmarried	1.093(0.618-2.007)	0.502(0.140-1.875)	0.912(0.594-1.416)	0.762(0.209-3.180)
Cigarette smoking	Former/Non	1.114(0.383-2.580)	2.964(0.432-12.72)	2.016(1.070-3.544)	2.781(0.405-11.97)
	Current/Non	0.521(0.156-1.300)	1.629(0.235-7.074)	0.644(0.283-1.274)	1.676(0.243-7.256)
Current menstruation	Yes/No	0.614(0.273-1.450)	0.079(0.009-0.452)	0.702(0.374-1.363)	0.157(0.027-0.694)
Sexual activity	Yes/No	1.223(0.667-2.198)	0.486(0.068-2.181)	1.064(0.679-1.656)	0.147(0.008-0.838)
Current constipation	Yes/No	0.693(0.416-1.146)	1.078(0.316-3.839)	1.147(0.776-1.708)	1.604(0.469-6.292)
Habitual bidet toilet use	Yes/No	0.818(0.480-1.395)	0.507(0.127-1.794)	0.871(0.573-1.316)	0.870(0.235-3.117)

c. Vaginal infection and vulval pruritus

		Doctor's diagnosis of:			Subjective symptoms of:		
		Candida vaginitis	Bacterial vaginitis	Vulval pruritus	Candida vaginitis	Bacterial vaginitis	Vulval pruritus
Age distribution	Elder/Young	0.036(0.002-0.427)	0.046(0.002-0.515)	0.191(0.022-1.754)	0.515(0.073-10.41)	0.367(0.091-1.567)	0.427(0.179-1.055)
	Middle/Young	0.168(0.051-0.452)	0.371(0.087-1.374)	0.418(0.077-2.012)	0.326(0.145-0.672)	0.830(0.354-1.893)	0.736(0.397-1.339)
Marriage status	Married /unmarried	1.309(0.611-2.892)	0.611(0.198-1.980)	0.972(0.287-3.830)	0.562(0.306-1.029)	0.444(0.220-0.904)	0.670(0.416-1.089)
Cigarette smoking	Former/Non	1.419(0.327-4.296)	2.108(0.314-8.631)	3.790(0.538-17.85)	1.082(0.315-2.828)	1.717(0.568-4.242)	0.893(0.342-1.929)
	Current/Non	2.875(1.164-6.469)	3.044(0.790-9.992)	7.618(2.071-28.02)	1.735(0.790-3.492)	1.839(0.715-4.168)	1.292(0.654-2.351)
Current menstruation	Yes/No	1.597(0.404-10.70)	0.665(0.156-3.497)	0.341(0.066-1.993)	6.742(1.348-122.6)	1.331(0.488-4.256)	0.786(0.397-1.632)
Sexual activity	Yes/No	1.180(0.541-2.675)	1.873(0.576-6.807)	1.349(0.363-5.090)	1.720(0.922-3.271)	1.891(0.905-4.039)	1.109(0.668-1.828)
Current constipation	Yes/No	1.841(0.850-4.431)	0.982(0.334-3.051)	4.314(1.108-28.47)	1.403(0.785-2.603)	2.212(1.087-4.872)	1.951(1.221-3.205)
Habitual bidet toilet use	Yes/No	0.814(0.330-1.817)	2.679(0.866-8.678)	1.001(0.276-3.415)	1.325(0.706-2.424)	2.662(1.315-5.520)	0.803(0.491-1.298)

Supplemental Table 3 Characteristics of subjects analyzed and unanalyzed, and comparison of proportion to the National Survey data

	Subjects analyzed n(%)	Subjects unanalyzed[a] n(%)	Proportion (%) of National data[b]	Proportion (%) of Subjects analyzed	Δ
Use of bidet toilet					
Habitual	4,272(78.9)	1145(21.1)			
Non-habitual	3,365(68.8)	1,523(31.2)			
Gender					
Male	3,772(76.2)	1180(23.8)	48.7	49.4	-0.7
Female	3,865(72.2)	1,488(27.8)	51.3	50.6	0.7
Age distribution					
20's	716(54.0)	611(46.0)	14.1	9.4	4.7
30's	1,186(66.6)	594(33.4)	18.5	15.5	3.0
40's	1,261(76.3)	391(23.7)	17.9	16.5	1.4
50's	1,296(80.0)	325(20.0)	16.5	17.0	-0.5
60's	1,692(83.7)	329(16.3)	19.2	22.2	-3.0
70's	1,486(83.3)	298(16.7)	13.8	19.5	-5.7
Residential district[c]					
Hokkaido	363(71.0)	148(29.0)	4.4	4.8	-0.4
Tohoku	362(71.4)	145(28.6)	7.2	4.7	2.5
Kanto	3,186(75.4)	1,037(24.6)	33.7	41.7	-8.0
Chubu	1,110(75.3)	364(24.7)	18.2	14.5	3.7
Kinki	1,559(74.0)	548(26.0)	16.3	20.4	-4.1
Chugoku	356(72.5)	135(27.5)	5.9	4.7	1.2
Shikoku	174(71.3)	70(28.7)	3.1	2.3	0.8
Kyushu	527(70.5)	221(29.6)	11.2	6.9	4.3
Marriage status[d]					
Married	5,288(77.0)	1,582(23.0)	72.2	69.2	3.0
Unmarried	2,349(68.4)	1,086(31.6)	27.8	30.8	-3.0
Household income (million yen/yr)					
<4	2,474(73.3)	903(26.7)	48.2	37.5	10.7
4- <8	2,846(76.3)	884(23.7)	31.9	43.2	-11.3
>=8	1,273(76.7)	386(23.3)	19.9	19.3	0.6
Educational background					
Low	3,289(73.9)	1,160(26.1)			
High	4,298(74.3)	1,486(25.7)			
Cigarette smoking					
Never	5,073(73.9)	1,795(26.1)			
Former	1,224(78.7)	331(21.3)			
Current	1,340(71.2)	542(28.8)			
Alcohol drinking					
No	1,956(73.0)	722(27.0)			
<once/week	2,900(71.7)	1,144(28.3)			
>=once/week	2,781(77.6)	802(22.4)			
Frequency of showering/bathing					
>=once/day	5,880(73.4)	2,131(26.6)			
<once/day	1,475(76.8)	446(23.2)			
Current constipation					
Yes	3,429(72.7)	1,287(27.3)			
No	4,208(75.3)	1,381(24.7)			
Current menstruation					
Yes	1,630(65.5)	860(34.5)			
No	2,222(78.1)	624(21.9)			
Direction of wiping anus after defecation					
Front to back	2,277(72.5)	864(27.5)			
Back to front	1,264(71.0)	516(29.0)			
Sexual activity (>=once/year)					
Yes	1,229(67.2)	601(32.8)			
No	2,407(75.5)	783(24.6)			

[a]Subjects loss-to-follow-up and removed from analyses. [b]Calculated from "Statics of Japan 2013" (Ministry of Internal Affairs and Communications. http://www.stat.go.jp/data/nihon/back13/02.htm)(2015.08.17) [c]Due to data format, the national data included population of 80 years old or above. [d]"Married" included bereaved husbands/wives and "Unmarried" included divorced husbands/wives.
